# Bilberry-containing supplements on severe dry eye disease in young and middle-aged adults: A 3-month pilot analysis

**DOI:** 10.3389/fnut.2023.1061818

**Published:** 2023-01-19

**Authors:** Wing Y. Yu, Lily Y. L. Chan, Aden Chung, Paul H. Lee, George C. Woo

**Affiliations:** ^1^School of Optometry, The Hong Kong Polytechnic University, Kowloon, Hong Kong SAR, China; ^2^Southampton Clinical Trials Unit, University of Southampton, Southampton, United Kingdom; ^3^Centre for Eye and Vision Research (CEVR), 17W Science Park, Hong Kong SAR, China

**Keywords:** dietary, extract, anthocyanins, fish oil, Ocular Surface Disease, phenol red thread test

## Abstract

**Purpose:**

To explore the effect of bilberry and fish oil combination supplement on a small clinical sample patient-base with severe dry eyes.

**Methods:**

Twenty-four subjects were recruited with twelve randomly assigned to the intervention and control groups, respectively. Inclusion criteria included severe dry eye symptoms determined by scores >33 from the Ocular Surface Disease Index (OSDI) questionnaire. The intervention group was instructed to take an oral supplement with key ingredients of 600 mg bilberry extract and 240 mg docosahexaenoic acid-refined fish oil once daily for 3 months. The control group did not take any supplements. Mean changes in OSDI score, non-invasive tear break-up time (NITBUT), phenol red thread test (PRT), and percentage of meibomian gland openings were used as outcome measures. Testing was done at baseline, 1-month, and 3-month follow-up. Comparison between the treatment and control groups, and the younger adult and middle-age groups were performed.

**Results:**

The mean baseline values for the treatment and control groups were not clinically different. The OSDI score, NITBUT, PRT, and percentage of meibomian gland openings improved after taking the supplements for 3 months. The OSDI score, NITBUT, and PRT showed clinical improvements between the intervention and control groups. These improvements were consistent between the two age groups.

**Conclusion:**

This study suggested preliminary improvements in signs and symptoms of severe dry eyes that were independent of age after taking dietary supplementation of bilberry extract and fish oil for 3 months. Further studies using more device-based measures and a placebo supplement are warranted.

## 1. Introduction

Dry eye disease (DED) is one of the most prevalent eye conditions affecting populations of middle age or above with a prevalence of 14 to 33% worldwide (1, 2). Accompanying symptoms depict one of the most frequent reasons for seeking medical consult from optometrists, rendering it a significant public health problem (3). According to the Dry Eye Workshop report, DED is a multifactorial disease affecting the ocular surface and can lead to discomforting symptoms affecting visual functions (4). It can be caused by a reduction of tear secretion due to lacrimal gland hypofunction, which is associated with oxidative stress and inflammation (5). Besides, meibomian gland dysfunction and ocular surface inflammation are often associated with DED. It is unlikely that DED will lead to blindness; however, in its severe stage, it may disrupt work productivity and quality of life affecting driving, reading, and even screen time productivity (6, 7). There are diverse risk factors that may lead to DED including age, female gender, smoking, patients on certain medications, poor dietary intake, and accompanying systemic diseases such as Sjogren’s syndrome, rheumatoid arthritis, or other autoimmune diseases (8).

Treatment of DED usually involves the use of artificial tears and punctal plugs if there is aqueous tear deficiency. Artificial tears are generally prescribed as a mainstay treatment as it is an over-the-counter product that is commonly available. However, especially for older adult patients using eye drop bottles, difficulty in aiming the drop properly into the eye, and handling of the bottle, will eventually lead to non-compliance. Ocular surface complications may even result due to reactions to drop preservatives and contamination of bottle tip. Other DED treatments such as warm compress, lid hygiene, lipid-based ophthalmic ointment, and antibiotics may be considered if there is meibomian gland blockage. Anti-inflammatory medication such as cyclosporine may also be prescribed to address any ocular surface inflammation (9). While essential oils have been used as home remedies for dry eyes, there is little evidence related to its effectiveness for the treatment of dry eye disease. In recent years, it has been shown that lifestyle interventions such as exercise (10, 11) and dietary habits can be effective in improving dry eye (12). Over the past decade, more attention has been paid on dietary supplementation such as vitamins A, B complex, C, E, and omega-3 fatty acids for the prevention and treatment of DED. Research results have shown positive effects of these dietary supplements on tear stability, tear volume as well as symptoms in general (13–16).

Bilberry (*Vaccinium myrtillus* L.) contains an abundance of anthocyanins, which are water-soluble pigments in plants giving the fruit a violet-blue color. Animal studies have shown positive effects of bilberry in maintaining intestinal gut health, including reducing the risk of acute and chronic colitis as well as anti-oxidative properties in suppressing stress in the intestines (17, 18). There have also been reports on the positive antioxidant effects of bilberry on cognitive functions (19) as well as cardiovascular disease (20). When administered in a purified high-dose anthocyanin form, it has shown improved visual functions of myopes suffering from asthenopia (21) as well as improved accommodation functions when taken in a yeast-fermented bilberry extract form (22). So far, no major side effects have been reported on its use as a dietary supplement.

Previous scientific reports on bilberry and dry eyes have shown encouraging short-term effects after a 1-month administration period (23, 24). These studies showed subjective improvements in symptoms, which offers potential application value for dry eye sufferers needing further evaluation. More investigation is needed on effective therapeutic dosing and duration before conclusive evidence can be reached on whether previously observed results are consistent across different age groups. The objective of this pilot study was to evaluate whether a longer-term treatment for 3 months with bilberry extract and fish oil supplement relieved any signs and symptoms of severe dry eye compared to no intervention. We hypothesized that dietary supplementation with bilberry and fish oil would alleviate the signs and symptoms of severe dry eye in both young and older adult age groups compared to age-matched control groups.

## 2. Materials and methods

This study was a prospective examiner-masked, parallel controlled pilot study where subjects with severe DED symptoms were randomly assigned to bilberry treatment and control groups. The study was approved by The Hong Kong Polytechnic University Human Ethics Sub-committee (HSEARS20120721001). Eligible subjects were recruited from open recruitment flyers posted around the university campus. Informed consent was obtained from all subjects prior to data collection.

Subjects with any systemic or autoimmune diseases, such as Sjogren’s syndrome, rheumatoid arthritis, etc., and those who were on systemic medications affecting tear secretions such as antidepressants, anti-anxiety medications, antihistamines, diuretics, or pain medications that can affect ocular pain or discomfort sensation, with history of ocular surgery or currently active eye diseases, currently undergoing dry eye intervention, past/current smokers, and/or contact lens wearers were excluded from this study.

A total of 24 subjects were recruited in this study with 12 subjects randomly assigned to the intervention group and 12 subjects to the control group. Inclusion criteria included severe dry eye symptoms as determined by scores of >33 from the Ocular Surface Disease Index (OSDI) symptoms screening questionnaire (25).

### 2.1. Dry eye evaluation

Standardized tests and forms were used to evaluate both objective and subjective symptoms of DED. Mean changes in OSDI score, the non-invasive tear break-up time (NITBUT), phenol red thread test (PRT), and percentage of meibomian gland openings were used as outcome measures. Testing was done at baseline, 1-month, and 3-month follow-up.

### 2.2. Subjective test

Subjects first completed a subjective evaluation by completing an in-person interview-based OSDI questionnaire (Allergan, Inc., Irvine, CA, USA) to quantify the degree of dry eye symptoms. The examiner transcribed their replies on the OSDI form and tallied their scores according to the instructions given on the form. Details of the scoring can be assessed from: https://refresh.com.hk. Briefly, OSDI scores could range from 0 to 100, with higher scores indicating greater severity of symptoms. The score for diagnosis of mild DED is from 13 to 22. Subjects were classified as sufferers of moderate DED with a score from 23 to 32 and severe DED with a score ≥33 (25). The score obtained from the initial visit was used as the baseline score. The same procedure was repeated at follow-up and used as the post-score for comparison.

### 2.3. Objective tests

The TearScope (Oculus Keratographer) was used to evaluate NITBUT (timed interval in seconds) under a slit lamp according to the manufacturer’s instructions with an average of three readings recorded for each eye. The PRT (Zone-QUICK, Showa Yakuhin Kako Co., Ltd., Japan) was used to evaluate tear volume sufficiency according to the manufacturer’s instructions. The average based on two readings was recorded for each eye, with a 1-min break in between each measurement. It was used instead of Schirmer’s test since it was less invasive and showed less variation and higher sensitivity; thus, minimizing any reflex tearing and discomfort from distorting actual results. No topical anesthetic was used in this procedure. The percentage of meibomian gland openings was evaluated under video-recorded slit lamp examination on both the upper and lower eyelids to evaluate signs and severity of evaporative dry eye. The number of blocked glands was counted by reviewing the video recordings at baseline and follow-up visits.

### 2.4. Intervention, dosage, and duration of intervention

Subjects from the intervention group were instructed to take the assigned daily oral supplement for 3 months and none for the control group. As recommended by the manufacturer, subjects took six capsules [equivalent to a 2760 mg dosage (with key ingredients: 600 mg bilberry extract (150 mg anthocyanin) and 240 mg docosahexaenoic acid (DHA)-refined fish oil)], once a day before breakfast. The nutritional information per serving size of one capsule (460 mg) is listed in Table 1.

**TABLE 1:** Nutritional information per serving size of 1 capsule (460 mg) of the oral supplement used in the study.

Energy	2.68 kcal
Protein	0.11 g
Total fat	0.2 g
− Saturated fat	0.0 g
− Trans fat	0.0 g
Total carbohydrates	0.13 g
− Dietary fibers	0.02 g
− Sugars	0.0 g
Sodium	0.4 mg

An information sheet and a short briefing session were given to each subject to educate and serve as a reminder on the importance of compliance. Telephone follow-ups were made on weeks 3, 7, and 11 to ensure no adverse effects were reported and to ensure subject compliance was maintained. It also served as an appointment reminder to ensure no lose to follow-up. On-site follow-ups were made before the 5th week, and at the end of the 12th week. The intervention and control groups were reminded not to start any other new supplements during the enrollment period. They were also instructed not to change any living, dietary, and exercise habits during this period. Their ocular condition was re-examined by the masked examiner during subsequent follow-up visits. All subjects were educated about their dry eye condition at the end of the study period and were advised to continue care at the Optometry Clinic of The Hong Kong Polytechnic University after the completion of the study period, if necessary.

### 2.5. Statistical evaluation

The young adult group was defined as those <30 years old and the older adult group as those >48 years old. The dry eye classification was recorded and studied. All data were investigated using Microsoft Excel (Ver. 16.65). Data from both eyes were evaluated, and results from the worse eye at the baseline visit were used for subsequent analysis. Summary statistics were presented as the mean ± standard deviation (SD). Primary analysis combined all data to calculate pre- and post-treatment effects, and treatment versus control sets. Secondary analysis calculated whether there was any difference in treatment outcomes between the young and older adult population groups. Calculation of Cohen’s d effect size was performed for all outcome measures due to the small sample size.

## 3. Results

A total of 24 subjects (*n* = 12; 8 male; mean age = 22.6 ± 1.5 years for the young adult group and *n* = 12; 4 male; mean age = 52.0 ± 3.4 years for the older adult group) participated in this study. All were diagnosed with severe dry eye symptoms. There was no loss to follow-up for either group with the intervention group all showing positive compliance in taking the supplement (intake rate = 100%). The subject demographics are summarized in Table 2. Only data from the 3-month follow-up visit was used in the final post-treatment analysis as the 1-month follow-up was intended as a compliance and monitor check, without negative side effects reported or observed.

**TABLE 2:** The demographic characteristics of all participants.

Group	Combined (Young + older adults) (*n* = 12)	Combined (Young + older adults) (*n* = 12)	Young adults (*n* = 6)	Young adults (*n* = 6)	Older adults (*n* = 6)	Older adults (*n* = 6)
	**Treatment**	**Control**	**Treatment**	**Control**	**Treatment**	**Control**
Sex (Ratio) Male:Female	7:5	5:7	5:1	3:3	2:4	2:4
Age (Years) Mean ± SD	37.3 ± 15.00	37.3 ± 14.50	22.8 ± 1.17	22.3 ± 1.70	51.8 ± 3.76	52.2 ± 3.02

Subjects from the treatment group were instructed to take the assigned daily oral supplement with key ingredients of 600 mg bilberry extract and 240 mg docosahexaenoic acid (DHA)-refined fish oil for 3 months. Subjects from the control group did not take any supplements. SD, standard deviation.

### 3.1. Comparison between treatment and control groups at baseline

At the beginning of the study, the mean baseline values for the treatment group were similar to the control group (mean values of all variables measured were clinically similar to each other). Combined analyzed results are presented hereafter (Table 3).

**TABLE 3:** Dry eye disease characteristics of participants at baseline and 3-month follow-up.

Group	Combined (Young + older adults)		Young adults		Older adults	
	**(*n* = 12)**	**(*n* = 12)**	**(*n* = 6)**	**(*n* = 6)**	**(*n* = 6)**	**(*n* = 6)**
**Time point**	**Baseline**	**3-month**	**Baseline**	**3-month**	**Baseline**	**3-month**
**Ocular Surface Disease Index (OSDI) scores**						
Treatment	60.5 ± 6.13	49.2 ± 11.0	61.2 ± 7.14	50.4 ± 9.81	59.9 ± 5.53	48.0 ± 12.84
Control	64.1 ± 6.79	63.1 ± 5.39	64.2 ± 8.54	61.5 ± 6.09	64.0 ± 5.34	64.8 ± 4.50
**Non-invasive tear break-up time (NITBUT) (s)**						
Treatment	3.9 ± 0.88	5.4 ± 1.32	3.3 ± 0.63	4.9 ± 1.27	4.6 ± 0.61	5.8 ± 1.30
Control	3.5 ± 0.81	3.7 ± 0.79	2.9 ± 0.58	3.1 ± 0.58	4.0 ± 0.61	4.3 ± 0.44
**Phenol red thread test (PRT) (mm)**						
Treatment	10.8 ± 2.62	15.8 ± 2.63	11.6 ± 3.46	14.2 ± 1.83	10.1 ± 1.36	17.3 ± 2.42
Control	9.5 ± 1.90	10.2 ± 1.70	9.9 ± 2.13	10.3 ± 1.86	9.2 ± 1.75	10.0 ± 1.67
**Percentage of meibomian gland openings (%)**						
Treatment	44.7 ± 9.56	55.4 ± 6.52	41.1 ± 8.72	54.2 ± 6.43	48.2 ± 9.02	56.7 ± 6.38
Control	47.2 ± 7.17	52.0 ± 9.37	43.3 ± 5.85	52.0 ± 9.04	51.1 ± 6.19	52.0 ± 9.68

All data were presented as mean ± standard deviation. OSDI scores >33 were considered severe dry eyes. NITBUT < 5s and PRT < 10 mm were considered dry values. There were approximately 75 meibomian glands in an eye. An increase in percentage of meibomian gland openings indicated an improvement.

### 3.2. OSDI questionnaire

Results for OSDI are summarized in Table 3. Overall, the treatment group showed clinical improvement in symptoms—the overall higher scores at baseline visit reflected greater dry eye symptoms severity when comparing baseline with post-treatment. This was also evident on the change in OSDI scores in-between treatment and control groups at 3 months. The observed improvements in pre/post scores between the two age groups were similar.

### 3.3. NITBUT

An improvement was noted between the pre- and post- treatment. The change in NITBUT values also showed a greater improvement after a 3-month treatment than that of the control group. Both age groups showed similar improvements in pre/post values.

### 3.4. PRT

An improvement was noted in pre-/post-measures of the treatment group as well as in between treatment and control group analysis. Both age group analyses showed improvements.

### 3.5. Percentage of meibomian gland openings

Improvement in meibomian gland openings was noted at 3-month post-treatment compared to pre-treatment for both the treatment and control groups.

### 3.6. Comparison between pre- and post-treatment effect

There was greater improvement in mean changes from baseline between the treatment group and the control group in OSDI score (−11.21 points vs. −0.95 points), NITBUT (1.42 vs. 0.12 s), PRT (4.92 vs. 0.63 mm) and percentage of meibomian gland openings (10.8 vs. 4.8%) after taking the oral supplements for 3 months. Based on Cohen’s d effect size, all outcomes have a large difference effect (dOSDI = 1.72; dNITBUT = 1.60 and dPRT = 5.84), where these differences will be significant even with minimal sample size.

### 3.7. Comparison between intervention and control groups

Out of the four variables measured, the OSDI score, NITBUT, and PRT showed notable improvements between the intervention and control groups with mean of scores passing the clinical dry eye cut-off values.

### 3.8. Comparison between young and older age groups

The pre- and post-treatment changes between the young and older adult age groups were similar for all variables tested.

## 4. Discussion

This study provided pilot outcomes on the effect a 3-month dietary supplementation of bilberry extract and fish oil on signs and symptoms in adults with severe DED and the effect was independent of age. Recent DED research on the use of dietary supplements as treatment has shown promising results. Omega-3 fatty acid has been found to be useful in ameliorating subjective symptoms of dry eye due to its anti-inflammatory properties (26). Lactoferrin (12), probiotics, and functional foods that contain astaxanthin (16) have also been found to be effective in improving dry eye, highlighting the importance of dietary interventions in dry eye patients. It has also been reported that honeybee nutritional secretions (royal jelly) intake can lead to increased tear volume (27). The intake of supplements containing a combination of lactoferrin and lactic acid bacterium WB2000 has been found to be effective in improving dry eye (28). It was postulated that these observed benefits may have been a result of a modulation of the microbiome in the immune system and leading to a reduction of oxidative stress (29–31).

Anthocyanins are colored water-soluble pigments that belong to the flavonoid group. They are found in high concentrations in berries and believed to have anti-oxidative and anti-inflammatory properties (32, 33), which may be useful in DED treatment. Kawabata et al. (34) reported effective relief of asthenopic symptoms with a combined fish oil (DHA 783 mg), bilberry extract (anthocyanins 59 mg), and lutein (17.5 mg) supplement taken daily for 4 weeks. However, their study only involved a young adult population (treated *n* = 11, control *n* = 9) and did not involve objective dry eye measurements. In our older adult dry eye group, clinical improvement was noted in subjective OSDI rating, NITBUT, PRT, and percentage of meibomian gland openings. PRT showed a great improvement in both within group as well as compared to the control group, reflecting an increase in tear volume. Compared to Kawabata and Tsuji (34), our dosage involved a greater amount of bilberry extract (150 vs. 59 mg anthocyanin) and less DHA-refined fish oil (240 vs. 738 mg).

Ozawa et al. (23) reported that bilberry extract supplements taken daily at 480 mg/day (less than the amount 600 mg used in this study) for 8 weeks improved objective and subjective parameters of eye fatigue, including dry eye sensation induced by video display terminal loads in subjects (treated *n* = 43, control *n* = 37). Interestingly, orally administered maqui berry extract (that is also rich in anthocyanins) was found to restore tear secretion capacity in a rat blink-suppressed dry eye model (35). Later, a randomized double-blind placebo-controlled clinical trial showed that participants who took 60 mg of maqui berry extract (equivalent to 21 mg anthocyanins) per day for 4 weeks had a significantly higher lacrimal fluid production by Schirmer’s test and reduction of ocular symptoms than the control group (*n* = 37) (36). This echoed with results from another randomized double-blinded placebo-controlled study using bilberry extract, where subjects who took 160 mg of bilberry extract daily for 4 weeks (treated *n* = 11; control *n* = 10) had significant improvement in tear volume attributable to increased antioxidant potential (24). However, this study did not incorporate any established dry eye questionnaires as a measurement of subjective symptoms and only Schirmer’s test was used to quantify tear volume.

More recently, it was found that an oral supplement containing a botanical combination of lutein (20–28 mg), zeaxanthin (2–2.8 mg), extracts of blackcurrant (167–233 mg), chrysanthemum (125–175 mg), and goji berry (125–175 mg) improved dry eye symptoms and tear secretion after 90 days of intake in individuals who used visual display units for >6 h per day (37). In addition, a 1-month oral intake of a combination of zinc (10 mg), L-carnitine (50 mg), extracts of elderberry (300 mg), blackcurrant (100 mg), and Eleutherococcus (50 mg) significantly improved symptoms related to computer vision syndrome and contrast sensitivity (38). As patients with severe dry eye were recruited for our study, the bilberry dosage taken (600 mg bilberry extract equivalent to 150 mg anthocyanin) leaned toward a higher dosage regimen compared to those concentrations reported in previous literature.

To our knowledge, this is the first study to evaluate the qualitative as well as quantitative effects of a combined oral bilberry extract and fish oil supplementation has on severe dry eyes. The OSDI scores showed clinical improvement in values for those in the treatment group by their 3-month follow-up. This is pertinent, as a limited case series, where the combined bilberry-containing oral supplement may highlight some clinically relevant treatment gains for both age groups in just 3 months as indicated by their self-reported OSDI symptom measures. The NITBUT provided a clinical value on lipid layer integrity of the tear film. The significant improvement implies that bilberry may have a naturopathic value of improving tear film stability for those suffering from DED. Results of PRT implied an improvement in aqueous layer of the tear film; hence, tear quantity. However, whether the contribution was largely an added effect of the combined 600 mg bilberry extract (150 mg anthocyanin) and 240 mg DHA-refined fish oil formulary or anthocyanin alone requires further investigation. More recent reports on higher concentrations of fish oil omega-3 fatty acid supplements alone (3000 mg) for 12 months did not show significantly better outcomes than their placebo group using olive oil (39). However, there have been heated debates on whether olive oil, being used as a control, was considered to be neutral in its effect on dry eye disease. Other studies with a shorter follow-up using lower concentrations [1000 mg (40) and 1500 mg (41)] have shown omega-3 fatty acids to be effective. Altogether, most dry eye studies which showed improvements applied at least 1000 mg of fish oil omega-3 fatty acid. The 240 mg DHA contained within the capsule used in this study may not have yielded a significant therapeutic advantage.

As age is one of the risk factors for dry eye, we explored whether there were differences in improvement between age groups. Both age groups showed similar changes, which may reflect the same efficacy of bilberry and fish oil supplements across age groups. However, with a small sample size, no conclusive assumptions can be made without more results. The improvement noted in tear stability and tear quantity without any adverse events reported for the duration of the study period shows a promising value in applying bilberry extract to complement currently available DED treatment. It is also promising to note that improvements were not limited to subjective findings alone. Changes in dry eye signs substantiated encouraging findings that this combined dosage contributed to reasonable improvements within a short period. Furthermore, anthocyanins can be easily excreted from the body system unlike fat-soluble vitamins such as vitamins A, D, and E, which may accumulate in the body system, leading to toxicity if taken in overabundance.

In this pilot study, the methodology had some shortcomings as the sample size was limited, and the control group was not administered with a placebo due to the scale of resources available. All outcomes had a large effect based on Cohen’s d effect size (d_OSDI_ = 1.72; d_NITBUT_ = 1.60 and d_PRT_ = 5.84), where an effect size of d = 0.2, 0.5, and 0.8 correspond to effect sizes of small, moderate, and large, respectively (42). A large effect size shows that the treatment effect will be statistically significant even with a minimal sample size. Our study design was a parallel group comparison instead of being double-blinded. Further multicenter double-blind clinical trials to study tear composition should be conducted in the future. To develop a full clinical picture, objective diagnostic tests on ocular surface assessment, tear osmolarity, interferometry, meibomography, and tear meniscus height should be performed to improve DED monitoring. Visual functions such as contrast sensitivity are also recommended to monitor DED severity in future studies.

## 5. Conclusion

Observed improvements in signs and symptoms on a case series of patients with severe dry eyes have been noted after a 3-month treatment period using supplements with bilberry extract and fish oil.

## Data availability statement

The raw data supporting the conclusions of this article will be made available by the authors, without undue reservation.

## Ethics statement

This study was approved by The Hong Kong Polytechnic University Human Ethics Sub-committee (HSEARS20120721001). The participants provided their written informed consent to participate in this study.

## Author contributions

LC, AC, and GW designed the research. AC performed the research. WY, LC, PL, and AC analyzed the data and drafted the manuscript. WY, LC, PL, AC and GW revised the manuscript. All authors commented on and edited the manuscript and approved the final version of the manuscript.
